# Deep learning inference of cell type-specific gene expression from breast tumor histopathology

**DOI:** 10.1038/s41698-026-01419-9

**Published:** 2026-04-16

**Authors:** Andrew T. Wang, Saugato Rahman Dhruba, Emma M. Campagnolo, Keluo Yao, Peng Jiang, Kun Wang, Danh-Tai Hoang, Eytan Ruppin, Eldad D. Shulman

**Affiliations:** 1 Cancer Data Science Laboratory, Center for Cancer Research, National Cancer Institute, Bethesda, MD USA; 2 Departments of Pathology and Laboratory Medicine and Computational Biomedicine, Cedars-Sinai Medical Center, Los Angeles, CA USA; 3 Translational Research Institute and Jim and Eleanor Randall Department of Surgery, Cedars-Sinai Medical Center, Los Angeles, CA USA

**Keywords:** Breast cancer, Cancer microenvironment

## Abstract

Cell type-specific gene expression from single-cell RNA sequencing (RNA-seq) is valuable for breast cancer precision oncology but available cohorts are still limited due to its high cost. Deconvolution methods infer cell type-specific expression from bulk RNA-seq at a lower cost, yet expenses and processing time of bulk RNA-seq are also non-negligible and limit their application too. To address these limitations, we developed *SLIDE-EX* (SLide-based Inference of DEconvolved gene EXpression), a deep-learning framework that predicts cell type-specific gene expression and abundances directly from routine breast cancer histopathology whole slide images (WSIs), using deconvolved bulk RNA-seq data as training labels. Trained on the TCGA-breast cohort and tested in cross-validation and on an independent cohort of 160 cases, *SLIDE-EX* robustly infers the expression of thousands of genes across 9 distinct cell types, performing best for cancer-associated fibroblasts and cancer cells. The abundance of these two cell types could also be robustly predicted, together with that of myeloid cells. The robustly predicted genes reflect key biological functions of their respective cell types. From a translational perspective, the inferred cell-type-specific expression profiles predict chemotherapy response more accurately than models based on direct prediction from the slides or from the inferred bulk expression in two independent cohorts. Going forward, *SLIDE-EX* is a generic approach that opens up possibilities for rapid, cost-effective cell type-specific gene expression inference in potentially any cancer type, further democratizing the characterization of the tumor microenvironment.

## Introduction

Breast cancer remains the most common cancer among women worldwide, with over 2 million new cases diagnosed annually^1^. This heterogeneous disease is classified into multiple subtypes, each with distinct biological features and clinical outcomes^2^. Despite advances in early detection and treatment, accurately predicting patient prognosis and response to therapy remains challenging, highlighting the need for more precise diagnostic and prognostic tools. In recent years, transcriptomic signatures have emerged as powerful tools in breast cancer precision oncology. These signatures provide crucial insights into tumor biology, helping to guide treatment decisions and identify patients who may benefit from specific therapies^3^. For example, the PAM50 gene expression assay measures the expression of 50 classifier genes to categorize breast cancer into molecular subtypes and predict clinical outcomes^4^. The Oncotype DX, one of the most widely used gene expression assay in clinical practice, generates a recurrence score that serves as a prognostic biomarker and predicts the benefits from chemotherapy in patients meeting specific diagnostic criteria^5–7^. Moreover, gene expression patterns have shown promise in identifying novel biomarkers and predicting response to both standard chemotherapy^8^ and targeted treatments^3^. These advances suggest that continued development and validation of gene expression signatures hold promise for improving patient stratification and treatment selection in clinical practice.

Beyond tumor bulk gene expression, the tumor microenvironment (TME) is emerging as crucial for patient stratification and treatment response prediction. It has been shown that the clustering and morphology of tumor-infiltrating lymphocytes, as well as the density and clustering of cancer-associated fibroblasts, are prognostic in invasive breast cancer^9^. Effective tools to study the various cellular states of different cell types include spatial transcriptomics and single-cell RNA sequencing (scRNA-seq), but their use remains limited due to high cost and scarcity^10^. A more cost-effective approach to identifying clinically relevant cellular states is extracting cell type-specific expression from bulk tumor gene expression samples through computational deconvolution methods, and several algorithms have been developed for this purpose^11–13^. Most deconvolution algorithms utilize reference single-cell signatures. CIBERSORTx applies support vector regression, using these signatures as features to infer cell type proportions and gene expression^11^. MuSiC incorporates cross-sample variation to refine predictions, adjusting references to better fit bulk data^12^. CODEFACS, used in this study, enhances deconvolution through iterative optimization, predicting more genes with higher accuracy^13^. However, the potential clinical application of these methods remains limited by their reliance on bulk RNA-seq assays, which are costly and have long turnaround times, thereby constraining the use of gene expression-based biomarkers for TME analysis and treatment response prediction.

Recent advances in digital histopathology have enabled the extraction of clinically significant information from tumor slides using machine learning and artificial intelligence techniques, leveraging rapid progress in deep learning-based computer vision^14^. The application of deep learning to whole-slide images (WSIs) of tissue stained with hematoxylin and eosin (H&E) has already demonstrated potential in inferring molecular features from histopathology images, including genetic mutations^15^, tissue-level mRNA expression (bulk or spatially aggregated)^16,17^, and methylation patterns^18^. H&E slides are widely available and routine during cancer screening and diagnosis, making them ideal for greater accessibility of predictive cancer tools. Previous work to predict transcriptomics from H&E images has centered on bulk expression^16,19–21^, and this framework has been successfully used to predict cancer treatment response from inferred bulk expression intermediates in independent, retrospective clinical trial datasets^16,22^. More recent methods predict expression at spatial spot resolution^17,23^, but individual spots contain mixed cell populations, limiting cell type-specific inference. Prediction of cell type-specific gene expression from H&E slides therefore remains unexplored. Given the importance of transcriptomic signatures and expression in breast cancer treatment strategies, as well as recent developments in deconvolution methods and AI-driven biomarker prediction, we aim to investigate two main research questions: (1) To what extent can we predict cell type-specific data—both gene expression and cell abundance—from slide images? (2) Given a cell type-specific predictor, to what extent can we use these predictions to infer clinically relevant phenotypes?

To address this need, we developed *SLIDE-EX* (SLide-based Inference of DEconvolved gene EXpression), a deep-learning framework trained on deconvolved bulk transcriptomics data that robustly predicts cell type-specific gene expression and cell type abundance from histopathology slides. Importantly, *SLIDE-EX* predicts cell type-specific gene expression profiles, not merely cell type identity and abundance. This molecular characterization is inaccessible through visual inspection of histopathology. The prevalence of breast cancer and availability of comprehensive datasets guided our development and optimization of *SLIDE-EX* for this disease. We proceed to provide an overview of *SLIDE-EX*’s framework, the study design, and the cohorts analyzed. We then study the ability to predict cell type-specific data, demonstrating robust predictive performance of the trained model with high gene coverage on an independent, unseen cohort. Next, we applied *SLIDE-EX* to three cohorts of HER2-negative and triple negative breast cancer (TNBC) patients treated with neoadjuvant chemotherapy. Using the predicted cell type-specific gene expression values from these cohorts, we apply our previously published approach, DECODEM^24^, originally developed to predict chemotherapy response from cell type-specific gene expression profiles deconvolved from measured RNA-seq data, to predict patient response from the *SLIDE-EX* predictions. We show that this framework can accurately predict chemotherapy treatment response using only histopathology slides as the initial input.

## Results

### Study overview

We developed *SLIDE-EX*, a computational pipeline to predict *cell type states* from H&E whole slide images (WSIs) of breast cancer tumors to enable analysis of TME patterns associated with chemotherapy response without requiring resource-intensive molecular profiling. These *cell type states*, which we define as cell type-specific gene expression and abundance, were predicted for nine cell types: B-cells, Cancer-associated Fibroblasts (CAFs), Cancer Epithelial (malignant), Endothelial, Myeloid, Normal Epithelial, Plasmablasts, Perivascular-like Cells (PVL), and T-cells.

**Fig. 1:**
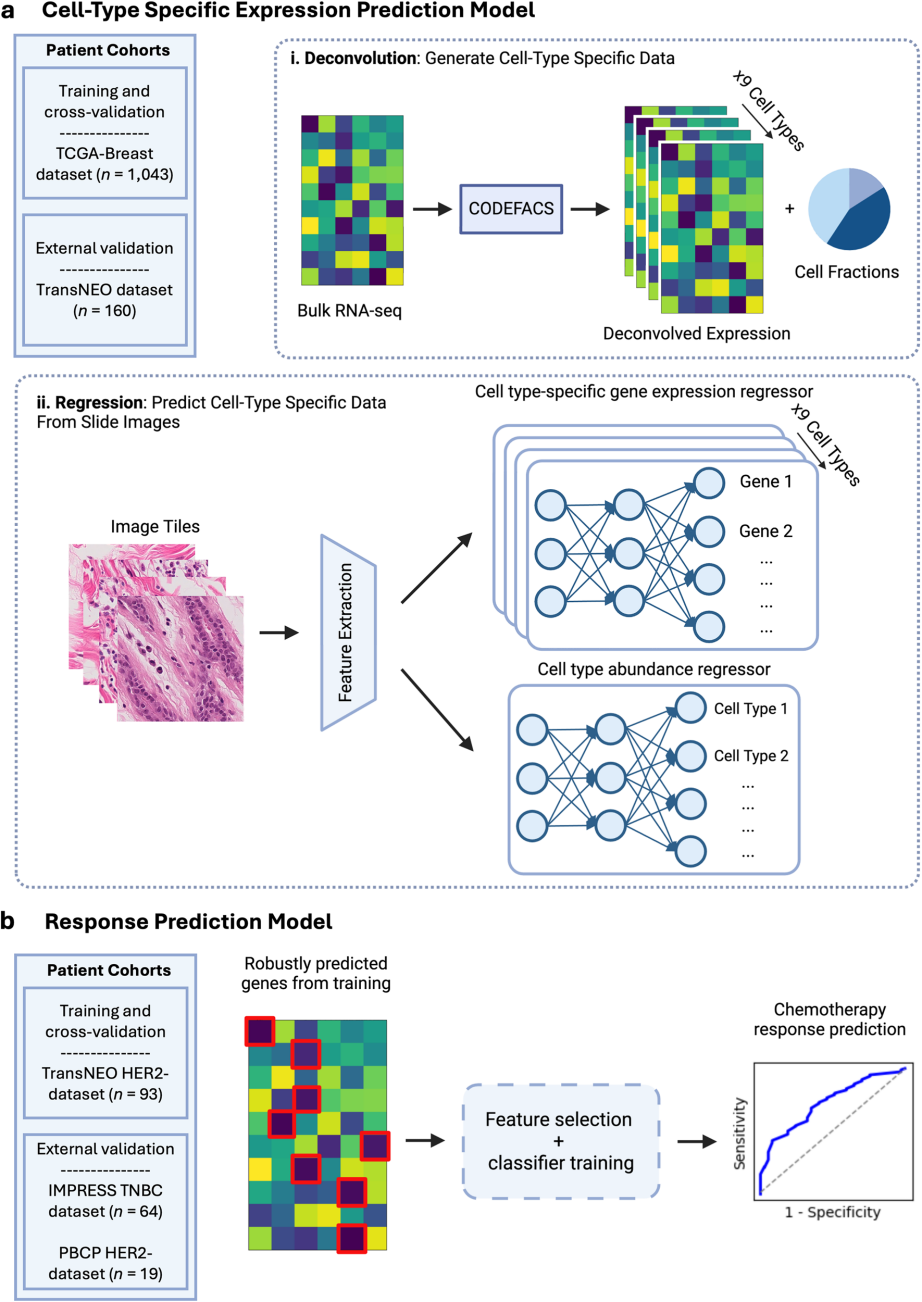
Overview of the datasets and computational workflow of *SLIDE-EX.* **a** The cell type deconvolution prediction model was trained and evaluated on datasets with paired H&E images and bulk transcriptomics from the TCGA-breast cohort. The TransNEO cohort was used for external validation. (**i**) The bulk transcriptomics data were deconvolved into cell fractions and cell type-specific gene expression using CODEFACS to serve as “ground truth” labels for the machine learning models. (**ii**) Whole slide images (WSIs) were divided into tiles and then color normalized, followed by feature extraction using the CTransPath digital pathology foundation model. The extracted features were then fed into a multi-layer perceptron (MLP) to infer cell type abundance and cell type-specific gene expression for each tile. **b** DECODEM was used to predict patient response to chemotherapy treatment based on the deconvolved gene expression predictions derived from (**a**). Training and cross-validation were performed on the TransNEO cohort, and the IMPRESS and PBCP cohorts were used for external validation. Only genes that were found to be well-predicted (PCC > 0.4) in the TCGA-breast cohort during training of the cell type-specific gene expression prediction task served as features for the DECODEM response prediction model. The response prediction classifier consists of an ensemble of logistic regression, random forest, and support vector machine models.

Using cell type states derived from CODEFACS-analyzed bulk RNA-seq data as ground truth, we developed ten deep learning models: one for cell type abundance and nine for cell type-specific expression. Each model employs image preprocessing with tile extraction, color normalization, and feature extraction using the digital pathology foundational CTransPath^25^, followed by a multi-layer perceptron (MLP) neural network for predicting cell type abundance or TPM normalized gene expression vectors (Fig. 1a). Importantly, SLIDE-EX does not explicitly identify or segment cell types within images. Rather, it learns statistical associations between histopathological patterns captured in H&E images (as represented compactly by a foundation model representation) and cell type-specific expression levels across patients through supervised regression. Cell type identity is defined entirely by the deconvolved expression profiles used as training labels, with no cell type annotations required in the images.

We chose CTransPath as the feature extraction module based on findings from our recently published Path2Omics study^26^. In that study, CTransPath outperformed UNI^27^, CONCH^28^, and Virchow2^29^ for predicting gene expression from H&E slides, with the largest performance advantage observed in the TransNeo-BRCA cohort. Because SLIDE-EX uses an identical feature extraction pipeline, differing only in training labels, this comparison applies directly to the current study.

We validated these models through cross-validation on 1043 TCGA breast cancer patients’ WSIs with matched RNA-seq data and assessed generalizability using the TransNEO cohort (*n* = 160). Prediction accuracy was further supported by multiple orthogonal lines of evidence, including significant overlap of well-predicted genes between cohorts, independent validation against directly measured single-cell expression, biological concordance of enriched pathways for each cell type, and demonstrated downstream clinical utility.

Specifically, to evaluate clinical utility, we investigated whether cell type-specific gene expression inferred by *SLIDE-EX* could predict neoadjuvant chemotherapy response using DECODEM, our computational framework for predicting chemotherapy response from deconvolved gene expression profiles (Fig. 1b). We trained and cross-validated the response prediction on the HER2-negative subset of the TransNEO cohort (*n* = 93) and validated it on the IMPRESS cohort^30^ (*n* = 64) and the HER2-negative subset of the Personalized Breast Cancer Program (PBCP) cohort (*n* = 19)^3^.

### Histopathology reveals cell type-specific gene expression patterns

**Fig. 2:**
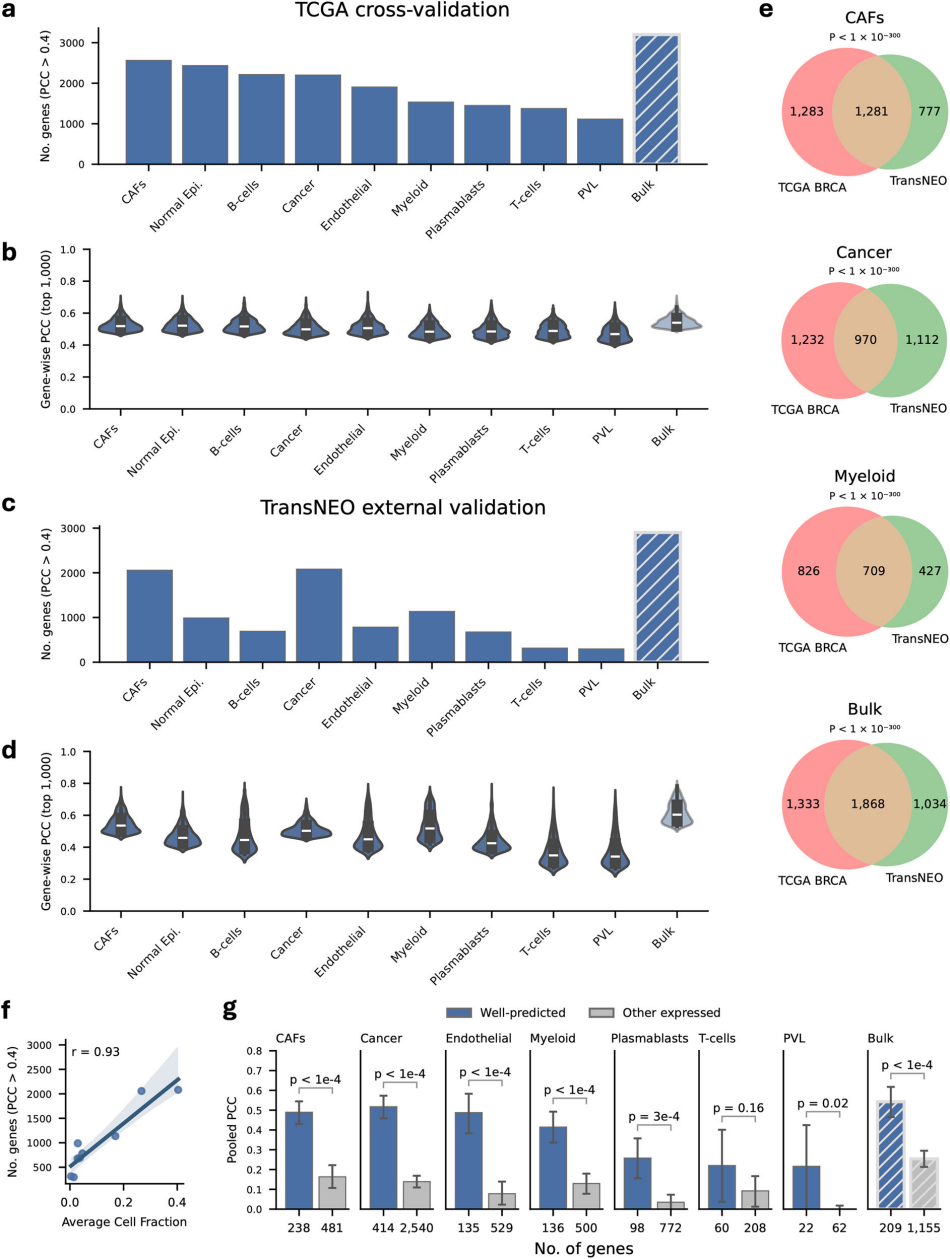
Performance of cell type-specific gene expression prediction from H&E slides. **a**, **b** Bar plots of the number of genes with a PCC of > 0.4 (**a**) and violin plots depicting the distribution of correlations for the top 1000 genes with the highest correlations (**b**) in the TCGA-breast cohort (cross-validation). PCCs are calculated between predicted and deconvolved expression values across the cohort for all differentially expressed genes for each subtype. Horizontal lines in violin plots represent median values. **c**, **d** Bar plots of the number of genes with a PCC of > 0.4 (**c**) and violin plots depicting the distribution of correlations for the top 1000 genes with the highest correlations (**d**) in the TransNEO cohort (external validation). **e** Venn diagrams illustrating the overlap between the well-predicted genes (PCC > 0.4) in TCGA-breast (left) and TransNEO (right) for the top 3 performing cell types in the external validation, as well as for the inferred bulk expression. *P*-values displayed above each plot were computed using a two-sided hypergeometric test to assess the statistical significance of gene overlap. **f** Scatterplot and linear regression of the number of well-predicted genes (PCC > 0.4) versus the average cell fraction per cell type across the TransNEO external validation cohort (*P* < 0.001, two-sided t-test). **g** Pooled Pearson correlations between SLIDE-EX-predicted and scRNA-seq-measured cell type-specific expression in three breast cancer patients from the MOSAIC dataset. Blue: well-predicted genes (PCC > 0.4 in TCGA); gray: other expressed genes. Error bars represent 95% confidence intervals from bootstrap resampling (*n* = 1000 replicates). *P*-values: permutation test (10,000 permutations). PVL ‘Other expressed’ truncated (Pooled PCC: −0.16; lower CI: −0.33).

As outlined in the overview, *SLIDE-EX* predicts cell type-specific gene expression profiles and cell type abundances directly from histopathology images for each of nine cell type categories. Here, we examine the performance of the expression models trained and evaluated using five-fold cross-validation on the TCGA breast cancer cohort. For each cell type, we excluded genes with low expression levels and low variance for each cell type, resulting in predictions for between 11,541 and 16,435 across the nine cell types (Methods). Model performance was evaluated using the Pearson correlation coefficient (PCC) between the predicted and deconvolved cell type-specific expression values of each gene, computed across all patients in the test set. This metric quantifies, for each gene, how well histopathological features predict its cell type-specific expression levels across the cohort. The majority of genes (>97% for all cell types) exhibited a positive correlation in the cross-validation of the TCGA-breast cohort (Supplementary Table 1, Supplementary Fig. 1a). Notably, for each of the nine cell types, *SLIDE-EX* predicted at least 1000 genes with a PCC greater than 0.4, with four cell types—CAFs, Normal Epithelial, B-cells, and Cancer Epithelial—exceeding 2000 genes above this threshold (Fig. 2a). The top-performing cell type predictor was for CAFs, which predicted 2564 genes with a PCC above 0.4, compared to 3201 genes predicted by a bulk gene expression predictor trained on the same computational framework. Among the top 1000 genes with the highest correlations, the averaged median PCC across the nine cell types reached 0.50, ranging from 0.47 (for PVL) to 0.52 (for CAFs, Normal Epithelial, and B-cells) (Fig. 2b).

To assess model generalizability, we applied the cell type-specific gene expression prediction models trained on the TCGA-breast dataset on the TransNEO cohort, an external validation dataset of 160 patients with matched tumor H&E WSIs and gene expression data. Notably, SLIDE-EX demonstrated robust performance despite substantial differences in sample preparation—TCGA slides are FFPE surgical resections whereas TransNEO slides are fresh-frozen core needle biopsies, preparation methods that produce visually distinct histological features (Supplementary Figs. 2,3). Across all cell types, the averaged median PCC for the top 1000 genes was 0.45, ranging from 0.34 for PVL to 0.54 for CAFs (Fig. 2d). Furthermore, the CAF and cancer epithelial cell predictors each achieved over 2000 well-predicted genes (PCC > 0.4) (Fig. 2c).

Well-predicted genes showed significant overlap between the training and external validation cohorts for each cell type (*P* < 10^−^^80^, two-sided hypergeometric test), indicating strong model consistency across datasets. In the TransNEO cohort, 62% of well-predicted CAF genes and 47% of well-predicted Cancer Epithelial genes overlapped with their respective well-predicted gene sets in TCGA-breast, comparable to the 64% overlap observed for bulk gene expression (Fig. 2e).

Prediction accuracy varied across cell types and exhibited a strong positive correlation with cell type abundance in the TransNEO external validation cohort (Pearson *r* = 0.93; Fig. 2f), with reduced performance observed for less abundant populations, consistent with prior reports for CODEFACS^13^. Stratification by clinical subtype further revealed significantly lower prediction performance in TNBC compared to HR-positive and HER2-positive tumors across all cell types (paired Wilcoxon signed-rank test, *P* < 0.001; Supplementary Fig. 4).

To evaluate SLIDE-EX predictions against ground truth independent of deconvolution, we compared model outputs to single-cell RNA sequencing data from the MOSAIC^31^ dataset, which includes matched H&E images and scRNA-seq profiles from breast cancer patients. Because only three patients were available, we assessed prediction accuracy for each cell type using a pooled Pearson correlation between SLIDE-EX-predicted and scRNA-seq-measured expression across all genes and patients (Methods). Across evaluable cell types, well-predicted genes exhibited substantially higher pooled correlations with single-cell ground truth than other expressed genes (Fig. 2g). For cancer epithelial cells and CAFs, the two cell types with the highest prediction performance in bulk cohorts, pooled correlations reached 0.52 (*n* = 414 genes) and 0.49 (*n* = 238 genes), respectively, compared to 0.14 and 0.16 for other genes (permutation test, both *P* < 10^−^^4^). Similar differences were observed for endothelial cells (0.49 vs. 0.08; *P* < 10^–4^) and myeloid cells (0.41 vs. 0.13; *P* < 10^−4^). Notably, these gene sets were defined entirely using bulk RNA-seq comparisons, yet the same genes showed stronger agreement with independently measured single-cell expression. A separate bulk expression model, trained and evaluated without deconvolution, achieved a pooled correlation of 0.54 (*P* < 10^−4^), comparable to the best-performing cell type-specific models (0.49–0.52), indicating that deconvolution-derived training labels are sufficiently reliable for learning cell type-specific expression from histology. Together, these results demonstrate that SLIDE-EX predictions capture biologically meaningful cell type-specific expression variation, and that bulk-derived performance metrics reliably identify accurately predicted genes.

### Slide-level supervision yields spatially coherent cell type predictions

The preceding analyses validated SLIDE-EX abundance predictions against CODEFACS-derived cell fractions at the whole-slide level. To assess whether these slide-level signals translate into accurate spatial localization, we next sought orthogonal validation against independent, morphology-based ground truth. We leveraged two breast cancer samples with expert pathologists’ binary annotations of cell type presence or absence at defined tissue locations (55 μm diameter regions corresponding to Visium spatial transcriptomics spots; 10x Genomics)^32,33^. For comparison with the pathologist’s three-category annotation scheme, we mapped SLIDE-EX predictions as follows: cancer cells (Cancer Epithelial predictions), stromal cells (CAF predictions), and lymphocytes (sum of B-cell and T-cell predictions).

Notably, SLIDE-EX was trained using only slide-level supervision, with a single abundance value per cell type per whole-slide image, and without any spatially resolved labels or localization objectives. Rather than performing explicit cell detection or segmentation, the model learns associations between histomorphology patterns and overall cellular composition. Consequently, any spatial agreement with localized annotations arises purely from morphology–expression associations learned from annotations provided at the global slide level, via weakly supervised multi-instance learning. This analysis therefore, tests whether morphology–expression relationships learned at the slide level transfer to localized tissue regions at a substantially finer spatial scale than that used during training.

**Fig. 3:**
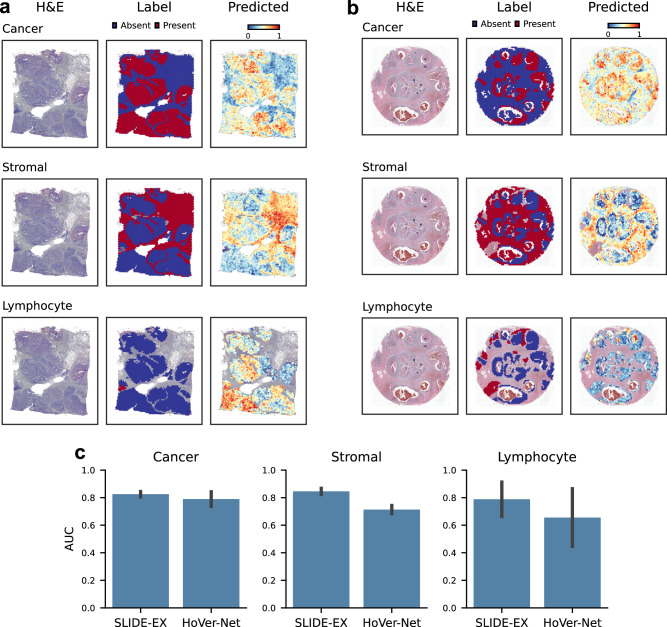
Validation of SLIDE-EX cell type abundance predictions against pathologist annotations at spatial resolution. **a**, **b** H&E images (left), binary pathologist annotations (middle; blue = absent, red = present), and SLIDE-EX predicted abundances (right) for cancer cells, stromal cells, and lymphocytes in two breast cancer samples: (**a**) TENX13 (*n* = 3775 regions) and (**b**) TENX39 (*n* = 2454 regions). Predictions were generated at 55 μm diameter tissue locations using a model trained with slide-level supervision only. Cancer, stromal, and lymphocyte predictions correspond to Cancer Epithelial, CAF, and summed B-cell plus T-cell abundances, respectively. **c** AUC values comparing SLIDE-EX to HoVer-Net, a nucleus-level cell detection model. AUCs were computed per sample and averaged; error bars show 95% bootstrap confidence intervals (*n* = 1000).

We applied the SLIDE-EX cell type abundance model to each annotated tissue region and compared predicted abundances against the binary pathologist annotations for three major cell types: cancer cells, stromal cells, and lymphocytes. Despite the absence of spatial supervision during training, visual inspection revealed strong spatial correspondence between SLIDE-EX predictions and pathologist annotations across both samples, with SLIDE-EX consistently predicting higher cell type abundances in regions annotated as containing the corresponding cell type (Fig. 3a, b). Across 6229 annotated regions from two samples (TENX13: *n* = 3775; TENX39: *n* = 2454), SLIDE-EX achieved AUCs of 0.82 for cancer cells, 0.85 for stromal cells, and 0.79 for lymphocytes for discriminating regions annotated as containing the cell type from those annotated as lacking it (Fig. 3c).

To contextualize these results, we compared SLIDE-EX to HoVer-Net^34^, a deep learning model explicitly designed for nucleus-level cell detection and classification in H&E images and trained with spatially resolved, cell-level supervision. Despite being trained solely with slide-level labels, SLIDE-EX outperformed HoVer-Net across all three cell types (HoVer-Net AUCs: 0.78, 0.71, and 0.65 for cancer cells, stromal cells, and lymphocytes, respectively; Fig. 3c). Together, these results indicate that SLIDE-EX captures morphological features that generalize across spatial scales, enabling accurate inference of local cell type composition without explicit spatial supervision.

### Histopathology images reveal cellular composition of the TME

**Fig. 4:**
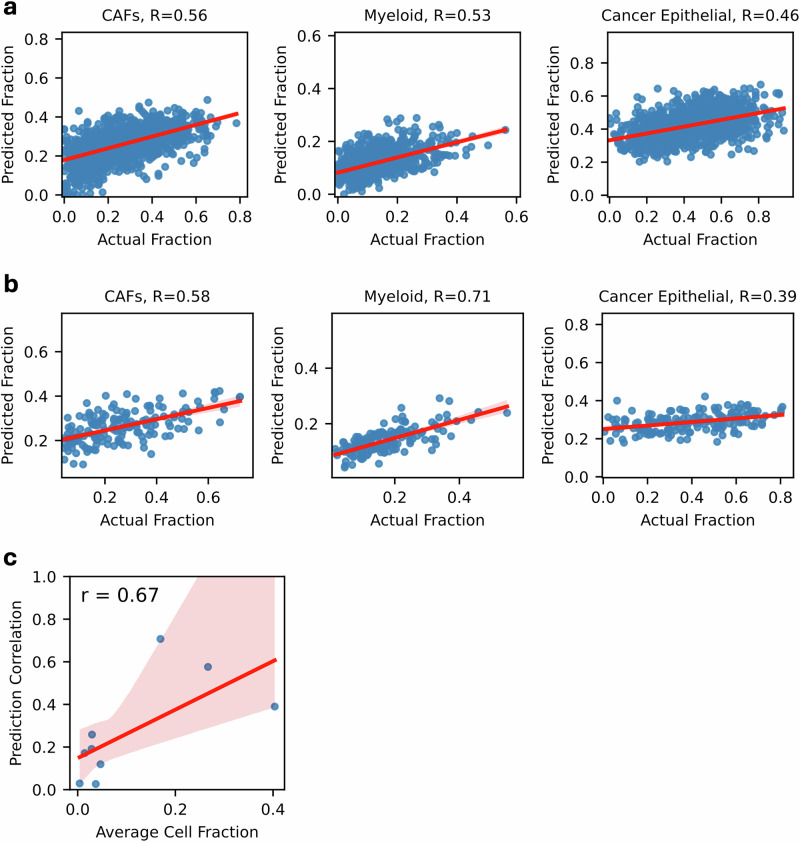
Performance of cell type abundance prediction from H&E slides. **a**, **b** Scatterplots of the predicted versus deconvolved fractions for the top 3 cell types with the highest PCCs in the TCGA-breast cross-validation cohort (**a**) and for the same 3 cell types in the TransNEO external validation cohort (**b**)**. c** Scatterplot and linear regression of the PCC between the predicted and deconvolved cell fractions versus the average cell fraction per cell type across the TransNEO external validation cohort (*P* < 0.05, two-sided t-test).

In parallel with predicting cell type-specific gene expression, we trained a model to predict the cell type fractions, or abundances, for the same nine cell types directly from histopathology image features in the TCGA-breast cohort. Using RNA-seq-derived cell type abundances as ground truth, we assessed model performance through PCCs between predicted and deconvolved cell fractions across all nine cell types in both training and validation datasets. During training and cross-validation, all cell types yielded positive correlations (*P* < 0.05 for all cell types, two-sided t-test), though prediction accuracy varied substantially across cell types (Supplementary Table 2). Three cell types achieved robust prediction performance (PCC > 0.4): CAFs (PCC = 0.56), Myeloid (PCC = 0.53), and Cancer Epithelial (PCC = 0.46) (Fig. 4a). To further validate the cell fraction predictor’s performance and demonstrate its generalizability, we again tested the model on the corresponding fresh-frozen H&E slides and deconvolved cell fractions of the TransNEO cohort. The top three performing cell types were the same in the independent validation, displaying PCCs above or around 0.4, and all cell types maintained a positive correlation. This suggests that our model can remain robust and generalizable across patient cohorts and tissue preparation methods. Surprisingly, the prediction correlation for Myeloid cells noticeably increased in the external validation compared to cross-validation, with a PCC of 0.71. The other two top cell types maintained similar performances to cross-validation (CAFs, PCC = 0.58; Cancer Epithelial, PCC = 0.39; Fig. 4b).

As with cell type-specific expression, *SLIDE-EX*’s ability to infer abundances is correlated with cell type abundances (Fig. 4c), highlighting that *SLIDE-EX* inferences for cell type fractions from WSIs are more robust for abundant cell types.

To assess the consistency of tile-level predictions within samples, we computed the coefficient of variation (CV = σ/μ) across tiles for each sample in the external validation cohort (TransNEO). For cell type abundance predictions, the median CV across samples was less than 0.5 for 6 of 9 cell types. For gene expression predictions, we first calculated CV for each gene within each sample, then summarized by taking the median across samples and then across genes; the resulting values ranged from 0.04 (T-cells) to 0.12 (Cancer Epithelial), with 8 of 9 cell types showing CV < 0.1 (Supplementary Table 3). These values indicate that tile-level predictions of both cell type-specific expression and cell type abundance are sufficiently consistent to support slide-level averaging.

### Robustly predicted genes reflect key biological functions of their respective cell types

**Fig. 5:**
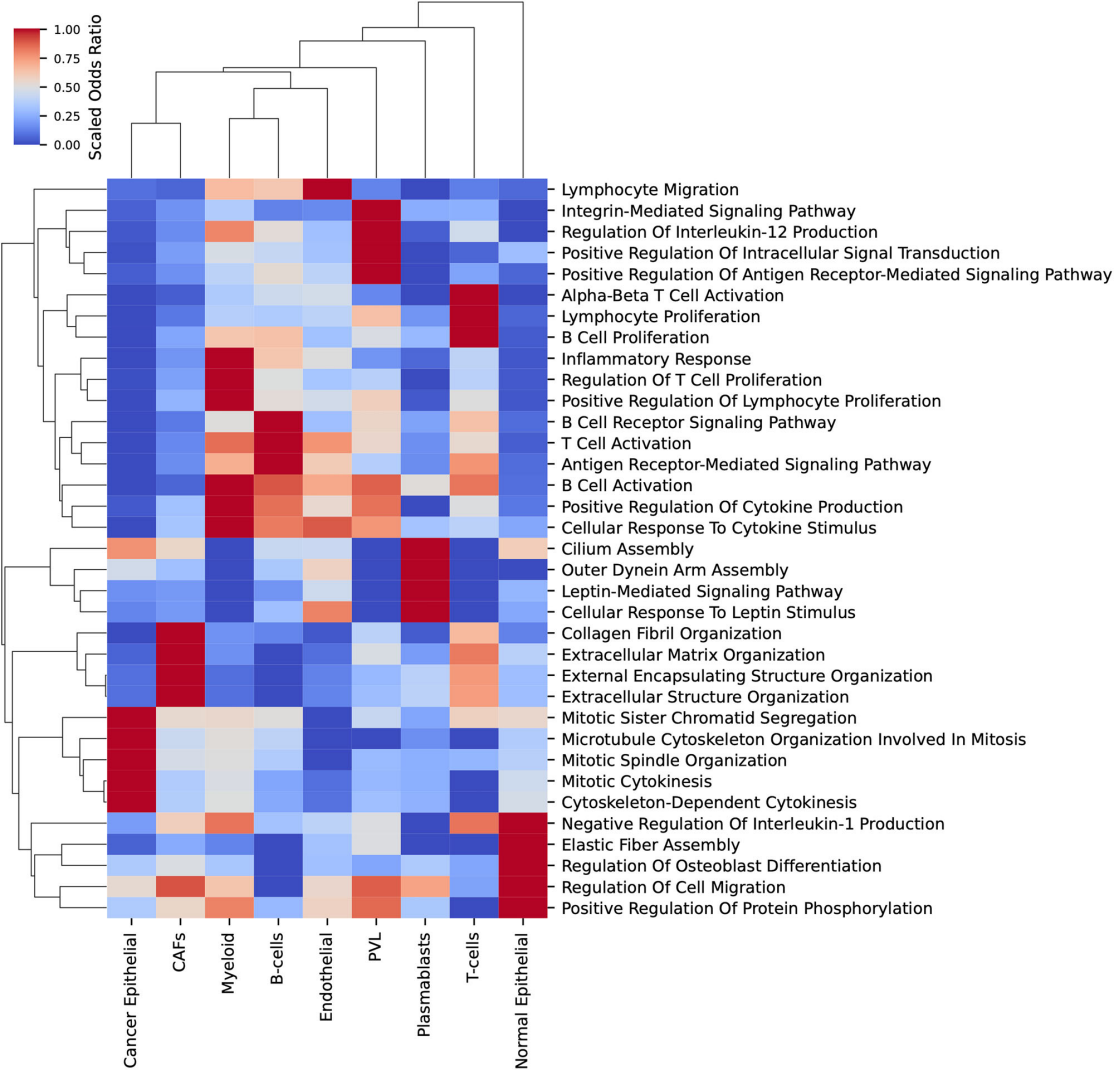
GO pathway enrichment analysis on the intersection of well-predicted genes (PCC > 0.4) in both cross-validation and external validation. The pathways displayed are the union of the top 5 most significantly enriched pathways for each cell type (hypergeometric test). Values (0–1) denote the odds ratios scaled per cell type (column).

We next explored whether genes that are robustly predicted (specifically having a PCC of >0.4 between predicted and deconvolved expression in both the TCGA-breast and TransNEO cohorts) have known biological relevance to cancer or to tumor immune microenvironment processes. To this end, we performed a Gene Ontology (GO) enrichment analysis on gene sets of the Biological Process aspect. Figure 5 summarizes the enrichment analysis results for the nine cell types of interest. Reassuringly, this analysis highlights the specific enrichment of cell cycle and DNA replication pathways among cancer cells. These are classical cancer hallmark pathways that are often disrupted in breast cancer^35–37^ and promote tumor proliferation, making them important prognostic markers and therapeutic targets^38–41^. Immune cells, on the other hand, exhibited enrichment in processes involved in immune response regulation, such as the activation and proliferation of T-cells, B-cells, and lymphocytes, and positive regulation of cytokine production. CAFs showed enrichment in pathways related to extracellular matrix organization and regulation of cell migration. These cell-type-specific enrichment patterns demonstrate that *SLIDE-EX* successfully captures genes central to each cell type’s biological function.

### The predicted cell type-specific gene expression predicts chemotherapy response

After establishing the predictive ability of these cell type-specific gene expression models, we sought to demonstrate their clinical utility by evaluating their ability to predict patient response to neoadjuvant chemotherapy. For this analysis, we utilized a subset of the TransNEO cohort, comprising 93 HER2-negative patients who received neoadjuvant chemotherapy and had available WSIs with corresponding response data. From these WSIs, we inferred cell type-specific gene expression for each of the nine cell types. Using the inferred gene expression from each cell type as features, we utilized DECODEM to develop cell type-specific machine learning classifiers to predict complete pathological response (pCR; *n* = 21). Each classifier was evaluated through cross-validation.

To benchmark our approach, we developed two baseline predictors: one utilizing inferred bulk expression values for classification, termed *inferred bulk*, and a second that classified patient response directly from image features by using the same computational pipeline as our expression predictors but replacing the regression head with a classification head. We termed this second approach *the direct supervised model*.

**Fig. 6:**
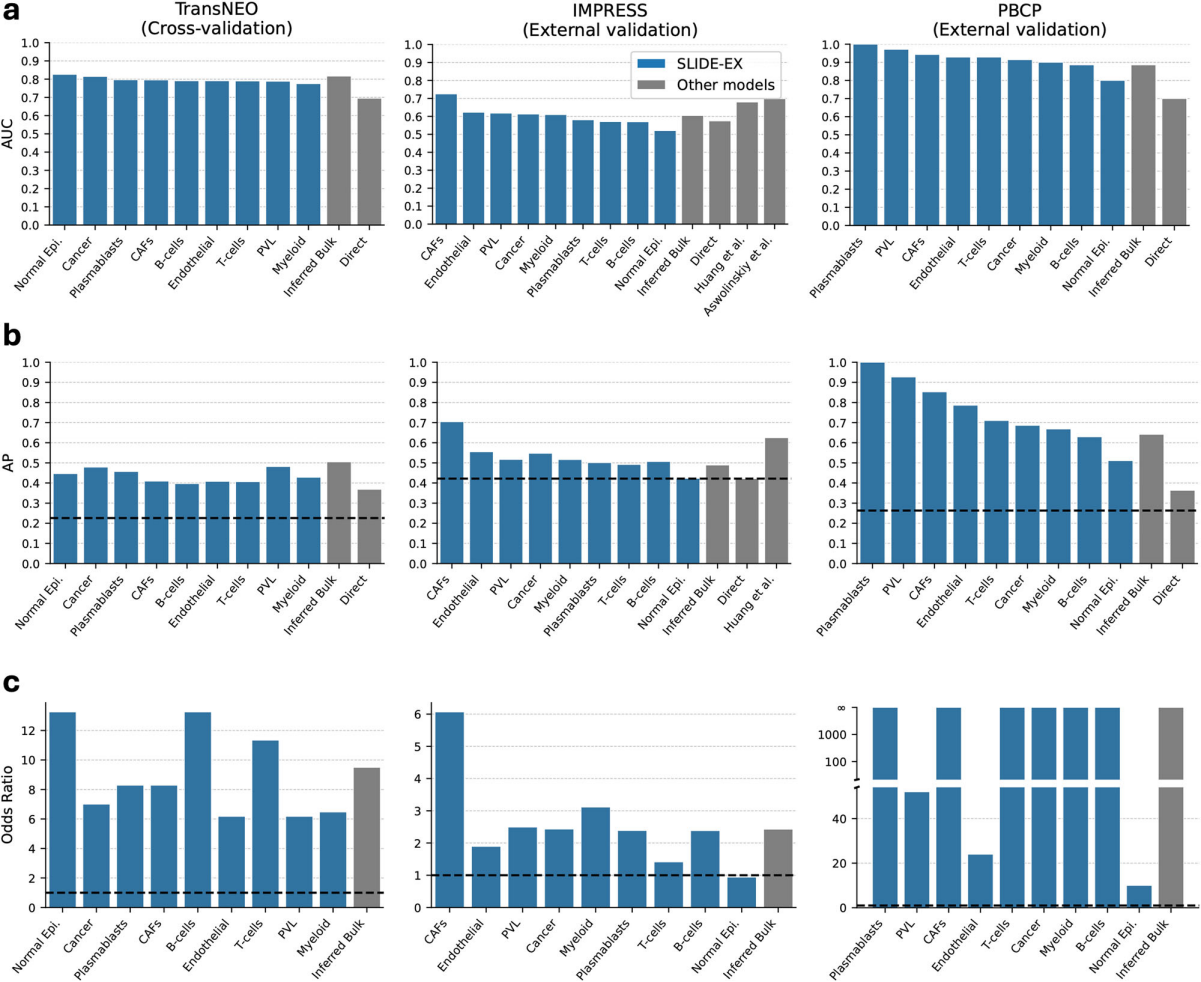
Predicting chemotherapy treatment response from H&E slides. **a** Bar plots of AUC values of response prediction for nine cell type-specific models compared to inferred bulk and direct predictors across three cohorts, with cell types ranked by descending values in cross-validation. Results shown for TransNEO HER2-negative cross-validation (*n* = 93; left), IMPRESS external validation (*n* = 64; middle), and PBCP external validation (*n* = 19; right). **b** Average precision (AP) values for all models across three cohorts, with dashed lines indicating each cohort’s overall response rate (ORR); An AP higher than the ORR demonstrates better-than-chance accuracy. Results shown for TransNEO HER2-negative cross-validation (*n* = 93; left), IMPRESS external validation (*n* = 64; middle), and PBCP external validation (*n* = 19; right). **c** Odds ratio values for all models across three cohorts, with dashed lines at OR = 1 indicating the threshold for positive association. Results shown for TransNEO HER2-negative cross-validation (*n* = 93; left), IMPRESS external validation (*n* = 64; middle), and PBCP external validation (*n* = 19; right).

In cross-validation on the TransNEO cohort, all nine cell type-specific models outperformed the direct model in terms of both area under the receiver operating characteristic curve (AUC) and average precision (AP; equivalent to the area under the precision–recall curve), and all exhibited similar performance to the bulk predictor (Fig. 6a, b; left). In addition, we observed a positive correlation between the prediction AUC of a cell type-specific model and the number of well-predicted genes (PCC > 0.4) for that cell type in the initial TCGA-breast cohort (Supplementary Fig. 5a, b).

To assess the generalizability and robustness of our models, we validated the predictive power of all cell type-specific models in external validation on two independent cohorts. We first used the IMPRESS cohort, comprising 64 TNBC patients who received neoadjuvant chemotherapy (*n* = 27 pCR) (Fig. 6a, b; middle). This validation was particularly challenging as models trained on TransNEO’s mixed subtypes were evaluated on IMPRESS’s exclusively TNBC cases. Notably, four cell type models achieved a higher test AUC than the bulk expression model. In particular, the CAF classifier reached an AUC of 0.72, compared to 0.60 for the bulk model (Fig. 6a; middle). For prediction performance assessed by AP, all cell type models except normal epithelial exceeded the overall response rate (ORR), reaffirming the predictive power of this pipeline (Fig. 6b; middle; dotted line). Importantly, our CAF-based model outperformed existing H&E-based methods for predicting chemotherapy response in breast cancer on the same challenging IMPRESS dataset, which reported AUCs of 0.68 (Huang et al.^30^; cross-validation) and 0.70 (Aswolinskiy et al.^42^; external validation). This demonstrates that cell type-specific transcriptional information derived from histopathology images can provide superior predictive power compared to conventional image-based approaches.

We then validated our models on a subset of the PBCP cohort, comprising 19 HER2-negative patients who received neoadjuvant chemotherapy (*n* = 5 pCR) (Fig. 6; right). This population closely resembled the HER2-negative patients we examined in the TransNEO cohort cross-validation. Notably, the Plasmablast classifier achieved perfect discrimination on the PBCP cohort, with both AUC and AP reaching 1.0. This finding aligns with clinical evidence that high densities of tumor-infiltrating plasmablasts and plasma cells, quantified through associated marker expression, predict chemotherapy response in breast cancer^43–45^. Furthermore, 8 of the 9 cell type-specific models demonstrated test AUCs exceeding or equal to the bulk model’s AUC of 0.89, and all cell type models surpassed the direct model (Fig. 6a; right). The APs attained by all cell type models substantially exceeded the ORR for the PBCP cohort, indicating robust predictive performance (Fig. 6b; right; dotted line).

To assess potential clinical applicability, we evaluated our models using the odds ratio (OR) of response. The OR quantifies the likelihood of response in patients predicted to respond compared to those predicted not to respond. Across all cell type-specific models, except for normal epithelial cells in IMPRESS, the odds were higher for predicted responders (OR > 1) (Fig. 6c). Notably, the CAF model, the top performing cell type in IMPRESS external validation, achieved an OR of 6.1, further indicating that patients classified as responders by our WSI-inferred cell type-specific expression models were more likely to achieve complete pathological response to chemotherapy, even in this challenging all-TNBC cohort (Fig. 6c; middle). In the PBCP cohort, 6 cell type-specific models, as well as the bulk model, achieved perfect separation, indicated by an OR of infinity (Fig. 6c; right).

To assess whether cell type-specific response classifiers provide information beyond established clinical prognostic factors, we evaluated their relationship with histologic grade, nodal status, and clinical stage in the TransNEO HER2-negative cohort. Univariate associations were consistent with known clinical patterns: higher histologic grade (Grade 3 vs. 2: OR = 5.68, *P* = 0.009), node-negative status (vs. node-positive: OR = 4.86, *P* = 0.003), and lower clinical stage (Stage II vs. III: OR = 3.91, *P* = 0.083) were each associated with increased likelihood of pCR^46,47^. To evaluate whether the cell type-specific response classifiers’ predictions were independent of these clinical variables, we performed a series of multivariable logistic regressions, one per cell type, with pCR regressed on four covariates: the cell type-specific classifier prediction, histologic grade, clinical stage, and nodal status. Seven of nine cell type-specific classifiers remained significantly associated with pCR (*P* < 0.05) after adjusting for the three clinical covariates, with the Normal Epithelial (*P* = 0.006), Myeloid (*P* = 0.005), and CAF (*P* = 0.009) classifiers demonstrating the strongest associations (Supplementary Fig. 6a). In these analyses, clinical covariates were no longer statistically significant (all *P* > 0.1), indicating that the cell type-specific expression predictions capture response-relevant information independent of histologic grade, clinical stage, or nodal status.

To further characterize classifier performance across clinical subgroups, we evaluated AUCs for classifying pCR within strata defined by histologic grade, nodal status, and clinical stage (Supplementary Fig. 6b). Predictive performance remained robust across strata, with a median AUC of 0.83 overall, including Grade 2 (median AUC = 0.91) and Grade 3 (median AUC = 0.68) tumors, node-negative (median AUC = 0.61) and node-positive (median AUC = 0.86) disease, and Stage II (median AUC = 0.74) and Stage III (median AUC = 0.93) patients. Stratified analyses were limited to the TransNEO cohort, as IMPRESS did not report histologic grade or clinical stage, and PBCP lacked stage information and exhibited limited grade heterogeneity (18 of 19 patients were Grade 3). More granular staging categories (e.g., IIA vs. IIB) were unavailable; nodal status was therefore analyzed separately to capture lymph node involvement.

## Discussion

This study presents *SLIDE-EX*, a deep-learning framework designed to predict *cell type states* (cell abundance and cell type-specific expression) directly from histopathology images. Prior methods have demonstrated that gene expression can be predicted from H&E images at either bulk resolution^16,19,26,48^ or spatial spot resolution^17,23,49^, but spots contain mixed cell populations. SLIDE-EX addresses a complementary problem: inferring expression profiles resolved by cell type rather than by spatial location. By leveraging deconvolution-derived training labels, SLIDE-EX enables prediction of expression for nine distinct cell types from routine histopathology, capturing transcriptional heterogeneity across the tumor microenvironment without requiring single-cell or spatial assays.

We first demonstrate its ability to predict deconvolved gene expression profiles and cellular fractions through cross-validation and external validation on tumor samples of various breast tumor subtypes. *SLIDE-EX*’s performance is robust over both bulk-derived and scRNA-seq-derived expression measurements. Remarkably, it also yields accurate spatial localization of cell types despite training on only slide-level labels.

Furthermore, we show that the cell type-specific gene expression profiles generated by *SLIDE-EX* predict chemotherapy response in three cohorts using only H&E images, outperforming existing approaches. By predicting chemotherapy response from routine H&E images available at diagnosis, this approach could enable earlier identification of patients unlikely to benefit from standard chemotherapy, potentially sparing them unnecessary toxicity and facilitating timely transition to alternative treatment strategies. Importantly, the robustly predicted genes for each cell type were enriched in Gene Ontology (GO) terms characteristic of the biological processes of the corresponding cell type, highlighting the biological relevance of *SLIDE-EX*’s predictions.

While our approach shows strong potential in predicting cell type-specific gene expression profiles directly from histopathology slide images, it has certain limitations. Notably, prediction performance was lower for less abundant cell types, likely due to their reduced representation in the images. Their limited presence may result in less distinguishable histological features, making it more challenging for the model to capture meaningful patterns associated with these cells. Additionally, our approach depends on the accuracy of cell type deconvolution from bulk RNA-seq data. We employed CODEFACS for deconvolution, and any limitations inherent to this method, such as variability in RNA-seq processing, reliance on well-defined molecular signatures, and a similar decrease in performance for less abundant cell types, can impact the downstream performance of *SLIDE-EX*. Another limitation of this study is that TCGA training paired FFPE histopathology sections with RNA-seq derived from fresh-frozen tissue, which are not always spatially adjacent. This spatial mismatch likely introduces noise during training and renders our reported performance a conservative estimate of what could possibly be achieved with spatially matched samples. Ultimately, *SLIDE-EX* provides an approximation of cellular states that can offer valuable biological insights and even predict treatment response in scenarios where resource-intensive single-cell or bulk RNA-seq data are unavailable. However, when SLIDE-EX is used to generate new biological hypotheses, these should be validated with single-cell or spatial transcriptomics, underscoring the continued importance of molecular assays for mechanistic discovery.

Overall, *SLIDE-EX* provides a cost-effective approach for predicting cell type-specific gene expression and cell type abundances, facilitating large-scale investigations that were previously constrained by limited access to transcriptomic assays. By enabling the exploration of gene expression patterns in patient cohorts lacking matched transcriptomic data, *SLIDE-EX* opens new avenues for uncovering key insights into the tumor microenvironment and its underlying molecular landscape in potentially many cancer types. This advancement lays the groundwork for more personalized treatment strategies and, ultimately, improved patient outcomes.

## Methods

### Data collection

All datasets used in this study are from publicly available sources. Links to download the datasets can be found in the Data Availability section below.

### *SLIDE-EX* computational framework

Our model architecture consists of four main components (Fig. 1a).

#### Deconvolution with CODEFACS

The first step involves employing CODEFACS, a cellular deconvolution tool recently developed in our lab^13^. We selected CODEFACS based on validated performance in breast cancer: in a benchmarking study across five breast cancer cohorts using pseudobulk samples with known single-cell ground truth, CODEFACS achieved high deconvolution accuracy and reliably inferred thousands of genes across cell types, supporting its use for generating training labels in the current study^24^. CODEFACS characterizes the TME by reconstructing the cell type-specific expression in each sample from the input measured bulk expression, in transcripts per million (TPM), utilizing the corresponding cell type abundance or a cell type-specific signature profile as another input. The output includes the cell type-specific expression profiles in TPM and the corresponding cell type abundances in each cell type across all samples.

In a previous work, we derived a cell type signature matrix of 1,400 genes representing nine cell types from a single-cell RNA-seq (scRNA-seq) cohort from Wu et al.^50^. We then deconvolved the bulk expression profiles with this signature using CODEFACS into cell type-specific expression profiles, encompassing nine cell types: B-cells, Cancer-associated Fibroblasts (CAFs), Cancer Epithelial (malignant), Endothelial, Myeloid, Normal Epithelial, Plasmablasts, Perivascular-like Cells (PVL), and T-cells.

#### Image processing

The preprocessing phase begins by dividing whole-slide images into non-overlapping tiles of 512 × 512 pixels at 20× magnification. Tiles that predominantly comprised more than half of the background were omitted. TCGA-BRCA slides are formalin-fixed, paraffin-embedded (FFPE) diagnostic sections from surgical resections; TransNEO and PBCP slides are fresh-frozen core needle biopsies; and IMPRESS slides are FFPE core needle biopsies (Supplementary Fig. 3). Color normalization was applied to mitigate staining variability across cohorts and preparation methods. In the TransNEO cohort, H&E whole-slide images and RNA-seq data were obtained from adjacent sections of the same fresh-frozen core needle biopsy, ensuring closer spatial correspondence between histopathological features and transcriptomic measurements than in TCGA.

#### Feature extraction

Image tiles were processed using CTransPath^25^, a foundational digital pathology model trained through self-supervision on histopathology images. Each tile is ultimately represented by a 768-dimensional CTransPath feature vector.

#### Multilayer perceptron regression

This component builds a predictive model linking CTransPath features to whole-genome gene expression at a cell type-specific level. The model architecture consists of three layers: (1) an input layer with 768 nodes, corresponding to the size of the CTransPath feature vector; (2) a hidden layer with 768 nodes; and (3) an output layer with one node per gene.

We selected this relatively simple MLP architecture for three reasons: (1) CTransPath already encodes rich histopathological features through self-supervised pretraining, so our objective was to learn a mapping from these pretrained features to expression values rather than to re-learn image representations; (2) simpler architectures reduce overfitting risk when predicting high-dimensional outputs (> 11,000 genes per cell type) from limited training samples; and (3) this architecture demonstrated strong performance for bulk expression prediction in our prior Path2Omics study^26^.

In order to facilitate convergence of our deep learning model, we excluded genes with low expression levels and low variance for each cell type, as well as genes that were not common to both the TCGA and TransNEO bulk expression profiles. Specifically, genes with non-zero expression in less than 50% of the cohort were excluded. In addition, genes with an expression level variance across the cohort of less than 10^−^^5^ were excluded. This threshold was determined empirically as the smallest variance that did not lead to interference with model convergence. Expression values were converted from TPM to logTPM, calculated as log(TPM + 1), before training. Since the deconvolved expression profiles varied from cell type to cell type, the number of genes predicted ranged from 11,541 to 16,435.

Because the training data consist of gene expression at the slide level (that is, bulk gene expression, as opposed to at spatial resolution), we averaged our per-tile predictions to obtain a mean value at the slide level. Alternatively, when predicting cell type abundance, the third and final layer instead has 9 nodes, one for each cell type. To ensure that the nine cell type abundance predictions are all non-negative and sum up to 1, the outputs are normalized by first subtracting the minimum value from the output vector if the minimum is negative, and then dividing each value by the sum of the output values.

### Model training and evaluation

To evaluate the performance of our models, we applied 5 × 5 nested cross-validation. For each outer loop, we split the entire patient population (of each cohort) into training (80%) and held-out test (20%) sets. We further split the training set into internal training and evaluation sets according to fivefold cross-validation. The models were trained and evaluated independently with each pair of training/validation sets. Averaging the predictions from the five different models represents our final prediction for each single gene and cell type on each held-out test set. We repeated this procedure five times across the five held-out test sets, making a total of 25 trained models. These models trained with the TCGA cohort were used to predict the expression of each gene and the abundance of each cell type in the external TransNEO cohort by computing the mean over the predicted values of all models.

The models were trained using stochastic gradient descent with a minibatch size of 32. For optimization, we employed the Adam optimizer with a learning rate of 10^−^^4^ for the cell type-specific expression models and 5 × 10^−^^6^ for the cell type abundance model. The mean squared error between predicted and deconvolved values was used as the loss function. To reduce the risk of overfitting, a dropout rate of 20% was applied to the initial layer. Each training round was conducted over 500 epochs. We also implemented early stopping to enhance computational efficiency and further prevent overfitting. The training was stopped if no improvement was observed in the mean correlation between predicted and deconvolved values on the validation set after 50 continuous epochs.

### Analysis of predicted cell type-specific gene expression

For downstream analysis of the predicted cell type-specific gene expression, we utilized averaged values from the 25 trained models in cross-validation as mentioned above.

Data on the cancer stage and subtype of each patient was obtained from the Genomic Data Commons Data Portal.

Gene Ontology (GO) enrichment analysis was performed using the Python package GSEAPY^51^ with the enrichr function for overrepresentation analysis. Gene sets were from the GO Biological Process aspect (The specific version is the ‘GO_Biological_Process_2023’ gene-set library from Enrichr)^52–54^. The cluster heatmap was plotted for the union of the top 5 enriched pathways of each cell type (Fig. 5).

### Validation against single-cell RNA sequencing

To validate SLIDE-EX predictions against measured cell type-specific expression, we utilized the MOSAIC^31^ dataset, which profiled breast cancer FFPE samples using Chromium Flex single-nuclei RNA sequencing alongside H&E whole slide imaging. Out of four breast cancer patients in that cohort, one was excluded due to low cell recovery (50 cells versus 1973–4729 in others), leaving three patients for analysis.

For each patient and cell type, pseudobulk expression profiles were computed by averaging normalized expression values across all cells annotated to that cell type. SLIDE-EX predictions were generated by applying models trained on TCGA-breast to the corresponding H&E images. Analysis was restricted to genes expressed in at least 20% of cells for each cell type.

Prediction accuracy was evaluated separately for each cell type using pooled Pearson correlations. For each gene, predicted and measured expression values were independently z-scored across patients. This standardization ensured equal contribution to the pooled correlation from each gene regardless of its mean expression level or variance. The resulting values were concatenated across all genes and patients within each gene set (well-predicted or other expressed). A single Pearson correlation between predicted and measured values was then computed within each gene set.

Confidence intervals (95%) were estimated via gene-level bootstrap resampling (1000 iterations). Statistical significance was assessed using a permutation test (10,000 permutations), in which gene labels were shuffled between well-predicted and other expressed groups to generate a null distribution of differences in pooled correlations.

### Validation against pathologist annotations at spatial resolution

To validate SLIDE-EX predictions against independent, morphology-based ground truth, we utilized H&E images from two FFPE breast cancer samples (TENX13 and TENX39) from 10x Genomics^32^ with expert pathologist annotations of cell type presence (cancer, stroma, lymphocyte) at defined tissue locations^33^. Annotations corresponded to Visium spot coordinates (55 μm diameter), providing 6229 annotated regions across the two samples (TENX13: *n* = 3775; TENX39: *n* = 2454).

For each annotated region, we extracted a single 300 × 300 pixel tile centered on the spot coordinates; this tile size was selected to avoid overlap with adjacent regions. The SLIDE-EX cell type abundance model trained on TCGA-BRCA was applied to each tile. To align SLIDE-EX’s nine-cell type output with the pathologist’s three-category annotation scheme, predictions were mapped as follows: cancer cells (Cancer Epithelial), stromal cells (CAFs), and lymphocytes (sum of B-cell and T-cell abundances).

Classification performance was assessed using the area under the receiver operating characteristic curve (AUC), treating pathologist annotations as binary ground truth (present vs. absent) for each cell type. AUCs were computed separately for each sample and then averaged. Confidence intervals (95%) were estimated via bootstrap resampling (*n* = 1000 replicates).

### Chemotherapy response prediction

DECODEM was used to predict patient response to chemotherapy treatment from the *SLIDE-EX* expression predictions. DECODEM takes in deconvolved gene expression profiles as input and trains an ensemble machine learning classifier as described in our previous work^24^. The ensemble consists of regularized logistic regression, random forest, and support vector machine models.

In the response prediction workflow, cell type-specific gene expression values were inferred via *SLIDE-EX* for each patient based on their corresponding H&E slide images. Then, these output expression predictions were fed into DECODEM. Of note, we included only genes that demonstrated robust prediction performance (PCC > 0.4) in the TCGA-breast cohort in cross-validation. This feature selection step was based on our hypothesis that well-predicted genes would provide stronger discriminating signals.

### Direct supervised model

The direct (end-to-end) supervised model was designed to classify responders and non-responders directly from their slides without the intermediate step of cell type-specific gene expression prediction. To this end, we applied the same computational deep-learning framework that was used for the prediction of gene expression, except that the MLP regression component was replaced by an MLP classification component. The same nested cross-validation approach was used to train and evaluate this model, except the TransNEO cohort was the cross-validation dataset and the IMPRESS cohort was the external validation dataset. For each model, slide-level prediction was computed by averaging tile-level predictions within that slide. A threshold of 0.5 was used to determine responder vs. non-responder from the model output.

### Clinical covariate analysis

To assess whether cell type-specific response classifiers provide predictive information independent of established clinical factors, we performed multivariable logistic regression in the TransNEO HER2-negative cohort. SLIDE-EX prediction scores for each cell type were binarized at the median into high and low groups. Clinical covariates considered included histologic grade, clinical stage, nodal status, and T stage. T stage was not associated with pCR in univariate analysis (*P* = 0.53) and was therefore excluded from subsequent models. Clinical stage was derived from T stage and nodal status according to AJCC staging criteria; patients with Stage I disease (*n* = 2) were excluded due to insufficient sample size. The final multivariable models included histologic grade (Grade 3 vs. Grade 2), clinical stage (Stage II vs. Stage III), and nodal status (node-negative vs. node-positive). For each cell type, a separate multivariable model was fitted with the binary SLIDE-EX score and all three clinical covariates as predictors. Odds ratios (ORs) and 95% confidence intervals (CIs) were estimated from model coefficients; *P*-values were derived from Wald tests.

To evaluate model performance within clinical subgroups, we computed AUC values for pCR prediction separately within strata defined by histologic grade (Grade 2, Grade 3), nodal status (node-negative, node-positive), and clinical stage (Stage II, Stage III).

### Implementation details

Analysis in this study was mainly performed in Python 3.9.7 with libraries including Numpy 1.20.3, Pandas 1.3.4, Scikit-learn 1.2.2, and Matplotlib 3.4.3. Image processing including tile partitioning and color normalization was conducted with OpenSlide 1.1.2, OpenCV 4.5.4 and PIL 8.4.0. The feature extraction, MLP regression, and MLP classification components were implemented using PyTorch 1.12.0.

## Supplementary information

Supplementary information

## Data Availability

FFPE histological images from the TCGA and their corresponding gene expression profiles are available in the Genomic Data Commons Data Portal (https://portal.gdc.cancer.gov). Fresh-frozen H&E-stained whole-slide images from the TransNEO and PBCP cohorts are available via Zenodo at: https://zenodo.org/records/6337925, and corresponding patient response data and RNA-seq counts can be accessed through the European Genome-phenome Archive at EGA: EGAS00001004582. FFPE whole-slide images and patient response data from the IMPRESS cohort are available at: https://tinyurl.com/IMPRESS-DATA. ScRNA-seq data and matched H&E whole-slide images from the MOSAIC cohort used for single-cell validation are available from the BioStudies database at: https://www.ebi.ac.uk/biostudies/arrayexpress/studies/E-MTAB-14560. Spatial transcriptomics data used for spatial validation were obtained from 10x Genomics at: https://www.10xgenomics.com/datasets.

## Code availability

The underlying code for this study is available and can be accessed via this link: https://zenodo.org/records/17727365.

## References

[1] Breast cancer. https://www.who.int/news-room/fact-sheets/detail/breast-cancer.

[2] Orrantia-Borunda, E., Anchondo-Nuñez, P., Acuña-Aguilar, L. E., Gómez-Valles, F. O. & Ramírez-Valdespino, C. A. Subtypes of Breast Cancer. in *Breast Cancer* 31–42 (Exon Publications, 2022). 36122153

[3] Sammut, S.-J. et al. Multi-omic machine learning predictor of breast cancer therapy response. *Nature* **601**, 623–629 (2022). 34875674 10.1038/s41586-021-04278-5 PMC8791834

[4] Pu, M. et al. Research-based PAM50 signature and long-term breast cancer survival. *Breast Cancer Res. Treat.* **179**, 197–206 (2020). 31542876 10.1007/s10549-019-05446-y PMC6985186

[5] Hacking, S. M., Yakirevich, E. & Wang, Y. From immunohistochemistry to new digital ecosystems: A state-of-the-art biomarker review for precision breast cancer medicine. *Cancers (Basel)* **14**, 3469 (2022). 35884530 10.3390/cancers14143469 PMC9315712

[6] Paik, S. et al. A multigene assay to predict recurrence of tamoxifen-treated, node-negative breast cancer. *N. Engl. J. Med.* **351**, 2817–2826 (2004). 15591335 10.1056/NEJMoa041588

[7] Kalinsky, K. et al. 21-gene assay to inform chemotherapy benefit in node-positive breast cancer. *N. Engl. J. Med.* **385**, 2336–2347 (2021). 34914339 10.1056/NEJMoa2108873 PMC9096864

[8] Barrón-Gallardo, C. A. et al. Transcriptomic analysis of breast cancer patients sensitive and resistant to chemotherapy: Looking for overall survival and drug resistance biomarkers. *Technol. Cancer Res. Treat.* **21**, 15330338211068965 (2022). 34981997 10.1177/15330338211068965 PMC8733364

[9] Amgad, M. et al. A population-level digital histologic biomarker for enhanced prognosis of invasive breast cancer. *Nat. Med.* **30**, 85–97 (2024). 38012314 10.1038/s41591-023-02643-7

[10] Smith, K. D., Prince, D. K., MacDonald, J. W., Bammler, T. K. & Akilesh, S. Challenges and opportunities for the clinical translation of spatial transcriptomics technologies. *Glomerular Dis.* **4**, 49–63 (2024). 38600956 10.1159/000538344 PMC11006413

[11] Newman, A. M. et al. Determining cell type abundance and expression from bulk tissues with digital cytometry. *Nat. Biotechnol.* **37**, 773–782 (2019). 31061481 10.1038/s41587-019-0114-2 PMC6610714

[12] Wang, X., Park, J., Susztak, K., Zhang, N. R. & Li, M. Bulk tissue cell type deconvolution with multi-subject single-cell expression reference. *Nat. Commun.* **10**, 380 (2019). 30670690 10.1038/s41467-018-08023-x PMC6342984

[13] Wang, K. et al. Deconvolving clinically relevant cellular immune cross-talk from bulk gene expression using CODEFACS and LIRICS stratifies patients with melanoma to anti-PD-1 therapy. *Cancer Discov.* **12**, 1088–1105 (2022). 34983745 10.1158/2159-8290.CD-21-0887 PMC8983586

[14] Rosenthal, J. et al. Building tools for machine learning and artificial intelligence in cancer research: Best practices and a case study with the PathML toolkit for computational pathology. *Mol. Cancer Res.* **20**, 202–206 (2022). 34880124 10.1158/1541-7786.MCR-21-0665 PMC9127877

[15] Coudray, N. et al. Classification and mutation prediction from non-small cell lung cancer histopathology images using deep learning. *Nat. Med.* **24**, 1559–1567 (2018). 30224757 10.1038/s41591-018-0177-5 PMC9847512

[16] Hoang, D.-T. et al. A deep-learning framework to predict cancer treatment response from histopathology images through imputed transcriptomics. *Nat. Cancer* **5**, 1305–1317 (2024). 38961276 10.1038/s43018-024-00793-2 PMC12413935

[17] Shulman, E. D. et al. AI-driven spatial transcriptomics unlocks large-scale breast cancer biomarker discovery from histopathology. *bioRxiv*. 10.1101/2024.10.16.618609. (2024).

[18] Hoang, D.-T. et al. Prediction of DNA methylation-based tumor types from histopathology in central nervous system tumors with deep learning. *Nat. Med.* **30**, 1952–1961 (2024). 38760587 10.1038/s41591-024-02995-8

[19] Schmauch, B. et al. A deep learning model to predict RNA-Seq expression of tumours from whole slide images. *Nat. Commun.* **11**, 3877 (2020). 32747659 10.1038/s41467-020-17678-4 PMC7400514

[20] Wang, Y. et al. Predicting molecular phenotypes from histopathology images: A transcriptome-wide expression-morphology analysis in breast cancer. *Cancer Res* **81**, 5115–5126 (2021). 34341074 10.1158/0008-5472.CAN-21-0482 PMC9397635

[21] Alsaafin, A., Safarpoor, A., Sikaroudi, M., Hipp, J. D. & Tizhoosh, H. R. Learning to predict RNA sequence expressions from whole slide images with applications for search and classification. *Commun. Biol.* **6**, 304 (2023). 36949169 10.1038/s42003-023-04583-x PMC10033650

[22] Dinstag, G. et al. Clinically oriented prediction of patient response to targeted and immunotherapies from the tumor transcriptome. *Med (N.Y.)* **4**, 15–30.e8 (2023). 10.1016/j.medj.2022.11.001 PMC10029756 36513065

[23] He, B. et al. Integrating spatial gene expression and breast tumour morphology via deep learning. *Nat. Biomed. Eng.* **4**, 827–834 (2020). 32572199 10.1038/s41551-020-0578-x

[24] Dhruba, S. R. et al. Enhanced prediction of breast cancer patient response to chemotherapy by integrating deconvolved expression patterns of immune, stromal and tumor cells. *Cancer Lett.* **641**, 218101 (2026). 10.1016/j.canlet.2025.218101 PMC12807300 41176212

[25] Wang, X. et al. Transformer-based unsupervised contrastive learning for histopathological image classification. *Med. Image Anal.* **81**, 102559 (2022). 35952419 10.1016/j.media.2022.102559

[26] Hoang, D.-T. et al. Path2Omics enhances transcriptomic and methylation prediction accuracy from tumor histopathology. *Cancer Res.* **85**, 5098–5112 (2025). 41166699 10.1158/0008-5472.CAN-25-4350 PMC12704547

[27] Chen, R. J. et al. Towards a general-purpose foundation model for computational pathology. *Nat. Med.* **30**, 850–862 (2024). 38504018 10.1038/s41591-024-02857-3 PMC11403354

[28] Lu, M. Y. et al. A visual-language foundation model for computational pathology. *Nat. Med.* **30**, 863–874 (2024). 38504017 10.1038/s41591-024-02856-4 PMC11384335

[29] Zimmermann, E. et al. Virchow2: Scaling self-supervised mixed magnification models in pathology. *arXiv [cs.CV]* (2024).

[30] Huang, Z. et al. Artificial intelligence reveals features associated with breast cancer neoadjuvant chemotherapy responses from multi-stain histopathologic images. *NPJ Precis. Oncol.* **7**, 14 (2023). 36707660 10.1038/s41698-023-00352-5 PMC9883475

[31] Dong, Y. et al. Transcriptome analysis of archived tumors by Visium, GeoMx DSP, and Chromium reveals patient heterogeneity. *Nat. Commun.* **16**, 4400 (2025). 40355415 10.1038/s41467-025-59005-9 PMC12069714

[32] Janesick, A. et al. High resolution mapping of the tumor microenvironment using integrated single-cell, spatial and in situ analysis. *Nat. Commun.* **14**, 8353 (2023). 38114474 10.1038/s41467-023-43458-x PMC10730913

[33] Ru, B., Huang, J., Zhang, Y., Aldape, K. & Jiang, P. Estimation of cell lineages in tumors from spatial transcriptomics data. *Nat. Commun.* **14**, 568 (2023). 36732531 10.1038/s41467-023-36062-6 PMC9895078

[34] Graham, S. et al. Hover-Net: Simultaneous segmentation and classification of nuclei in multi-tissue histology images. *Med. Image Anal.* **58**, 101563 (2019). 31561183 10.1016/j.media.2019.101563

[35] Molinari, M. Cell cycle checkpoints and their inactivation in human cancer: Cell cycle checkpoints and human cancer. *Cell Prolif.* **33**, 261–274 (2000). 11063129 10.1046/j.1365-2184.2000.00191.x PMC6496592

[36] Bower, J. J. et al. Patterns of cell cycle checkpoint deregulation associated with intrinsic molecular subtypes of human breast cancer cells. *NPJ Breast Cancer* **3**, 9 (2017). 28649649 10.1038/s41523-017-0009-7 PMC5445620

[37] Zhou, K., Sun, Y., Dong, D., Zhao, C. & Wang, W. EMP3 negatively modulates breast cancer cell DNA replication, DNA damage repair, and stem-like properties. *Cell Death Dis.* **12**, 844 (2021). 34511602 10.1038/s41419-021-04140-6 PMC8435533

[38] Repo, H. et al. A prognostic model based on cell-cycle control predicts outcome of breast cancer patients. *BMC Cancer* **20**, 558 (2020). 32546141 10.1186/s12885-020-07045-3 PMC7296704

[39] Vihervuori, H. et al. Varying outcomes of triple-negative breast cancer in different age groups-prognostic value of clinical features and proliferation. *Breast Cancer Res. Treat.* **196**, 471–482 (2022). 36261751 10.1007/s10549-022-06767-1 PMC9633490

[40] Thu, K. L., Soria-Bretones, I., Mak, T. W. & Cescon, D. W. Targeting the cell cycle in breast cancer: towards the next phase. *Cell Cycle* **17**, 1871–1885 (2018). 30078354 10.1080/15384101.2018.1502567 PMC6152498

[41] Zhang, J., Chan, D. W. & Lin, S.-Y. Exploiting DNA replication stress as a therapeutic strategy for breast cancer. *Biomedicines* **10**, 2775 (2022). 36359297 10.3390/biomedicines10112775 PMC9687274

[42] Aswolinskiy, W. et al. PROACTING: predicting pathological complete response to neoadjuvant chemotherapy in breast cancer from routine diagnostic histopathology biopsies with deep learning. *Breast Cancer Res* **25**, 142 (2023). 37957667 10.1186/s13058-023-01726-0 PMC10644597

[43] Schmidt, M., Micke, P., Gehrmann, M. & Hengstler, J. G. Immunoglobulin kappa chain as an immunologic biomarker of prognosis and chemotherapy response in solid tumors. *Oncoimmunology* **1**, 1156–1158 (2012). 23170262 10.4161/onci.21653 PMC3494628

[44] Alistar, A. et al. Dual roles for immune metagenes in breast cancer prognosis and therapy prediction. *Genome Med* **6**, 80 (2014). 25419236 10.1186/s13073-014-0080-8 PMC4240891

[45] Conte, B. et al. A 14-gene B-cell immune signature in early-stage triple-negative breast cancer (TNBC): a pooled analysis of seven studies. *EBioMedicine* **102**, 105043 (2024). 38447275 10.1016/j.ebiom.2024.105043 PMC10924177

[46] Cortazar, P. et al. Pathological complete response and long-term clinical benefit in breast cancer: the CTNeoBC pooled analysis. *Lancet* **384**, 164–172 (2014). 24529560 10.1016/S0140-6736(13)62422-8

[47] Spring, L. M. et al. Pathologic complete response after neoadjuvant chemotherapy and impact on breast cancer recurrence and survival: A comprehensive meta-analysis. *Clin. Cancer Res.* **26**, 2838–2848 (2020). 32046998 10.1158/1078-0432.CCR-19-3492 PMC7299787

[48] Pizurica, M. et al. Digital profiling of gene expression from histology images with linearized attention. *Nat. Commun.* **15**, 9886 (2024). 39543087 10.1038/s41467-024-54182-5 PMC11564640

[49] Zeng, Y. et al. Spatial transcriptomics prediction from histology jointly through Transformer and graph neural networks. *Brief. Bioinform.* **23**, bbac297 (2022). 10.1093/bib/bbac297 35849101

[50] Wu, S. Z. et al. A single-cell and spatially resolved atlas of human breast cancers. *Nat. Genet.* **53**, 1334–1347 (2021). 34493872 10.1038/s41588-021-00911-1 PMC9044823

[51] Fang, Z., Liu, X. & Peltz, G. GSEApy: a comprehensive package for performing gene set enrichment analysis in Python. *Bioinformatics* **39**, btac757 (2023). 36426870 10.1093/bioinformatics/btac757 PMC9805564

[52] Ashburner, M. et al. Gene ontology: tool for the unification of biology. The Gene Ontology Consortium. *Nat. Genet.* **25**, 25–29 (2000). 10802651 10.1038/75556 PMC3037419

[53] Gene Ontology Consortium et al. The Gene Ontology knowledgebase in 2023. *Genetics* **224**, iyad031 (2023). 36866529 10.1093/genetics/iyad031 PMC10158837

[54] Chen, E. Y. et al. Enrichr: interactive and collaborative HTML5 gene list enrichment analysis tool. *BMC Bioinforma.* **14**, 128 (2013). 10.1186/1471-2105-14-128 PMC3637064 23586463

