# Supplementary material for: Deep learning inference of cell type-specific gene expression from breast tumor histopathology
Source: NPJ Precis Oncol. 2026 Apr 16;10:311. doi: 10.1038/s41698-026-01419-9 (PMC13462871; doi:10.1038/s41698-026-01419-9)
Supplement: Supplementary file 1 [file 41698_2026_1419_MOESM1_ESM.docx]

## Supplementary information for:

## Deep learning inference of cell type-specific gene expression from breast tumor histopathology

#### Supplementary Tables

| Cell Type | Percent of genes with positive correlation |
| --- | --- |
| Normal Epithelial | 99.79 |
| B-cells | 99.79 |
| CAFs | 99.64 |
| Cancer Epithelial | 99.53 |
| Plasmablasts | 99.49 |
| Myeloid | 99.00 |
| PVL | 98.31 |
| Endothelial | 98.29 |
| T-cells | 97.98 |

**Supplementary Table 1: Percent of genes with a positive PCC for each of the nine cell types in the TCGA-breast cross-validation cohort.**

| Cell Type | Correlation on Cross-validation | Correlation on External Validation |
| --- | --- | --- |
| CAFs | 0.56 | 0.58 |
| Myeloid | 0.53 | 0.71 |
| Cancer Epithelial | 0.46 | 0.39 |
| Endothelial | 0.37 | 0.12 |
| B-cells | 0.35 | 0.03 |
| Plasmablasts | 0.32 | 0.19 |
| Normal Epithelial | 0.32 | 0.26 |
| T-cells | 0.22 | 0.03 |
| PVL | 0.07 | 0.17 |

**Supplementary Table 2:** **Pearson correlation coefficient between the predicted and deconvolved cell fractions for each of the nine cell types.** Cross-validation was performed on the TCGA-breast cohort, and external validation was performed on the TransNEO cohort. Cell types sorted by decreasing PCC on cross-validation.

| Cell Type | Median coefficient of variation of cell type abundance | Median coefficient of variation of cell type-specific gene expression |
| --- | --- | --- |
| B-cells | 0.58 | 0.06 |
| CAFs | 0.30 | 0.09 |
| Cancer Epithelial | 0.27 | 0.12 |
| Endothelial | 0.34 | 0.06 |
| Myeloid | 0.31 | 0.08 |
| Normal Epithelial | 0.36 | 0.07 |
| Plasmablasts | 0.46 | 0.06 |
| PVL | 0.88 | 0.04 |
| T-cells | 0.92 | 0.04 |

**Supplementary Table 3:** **Variability of tile-level SLIDE-EX predictions within samples.** The coefficient of variation (CV = σ/μ) across tiles for each sample in the TransNEO external validation cohort was computed. For cell type abundance, the median CV across samples was used as a measure of consistency of tile-level abundance predictions. For cell type-specific gene expression, the CV for the predicted expression level of each gene within each sample was calculated. Then, the extent of variability was summarized by first obtaining the median CV of each gene across samples, and then taking the median across genes.

#### Supplementary Figures

**Supplementary Fig. 1: Performance of cell type-specific gene expression prediction across all genes. a–b,** Violin plots depicting the distribution of correlations for all predicted genes (**a**) in the TCGA-breast cross-validation cohort and (**b**) in the TransNEO external validation cohort.

**Supplementary Fig. 2: UMAP projection of WSIs from TCGA and TransNEO cohorts in CTransPath embedding space.** For each WSI, the per-tile feature vectors from CTransPath were averaged to obtain a single mean embedding vector that represents the entire slide. The mean embedding vectors were then projected into UMAP space.

**Supplementary Fig. 3: Representative H&E whole slide images and extracted tiles from each study cohort.** **a–d,** Three representative WSIs are shown per cohort with sample identifiers: **(a)** TCGA-BRCA (FFPE surgical resections), **(b)** TransNEO (fresh-frozen core needle biopsies), **(c)** IMPRESS (FFPE core needle biopsies), and **(d)** PBCP (fresh-frozen core needle biopsies). Colored boxes indicate locations of extracted 512 × 512 pixel tiles shown below each WSI at 20× magnification. FFPE sections **(a, c)** exhibit well-preserved nuclear morphology and uniform staining, whereas fresh-frozen sections **(b, d)** show characteristic ice-crystal artifacts and variable staining intensity. These morphological differences illustrate the tissue preparation heterogeneity that SLIDE-EX must accommodate for cross-cohort generalization.

**Supplementary Fig. 4: Performance of cell type-specific gene expression prediction across breast cancer subtypes. a**, Box plots of the PCCs for the predicted genes across the breast cancer clinical subtypes in the TCGA-breast cohort. **b**, Bar plots of the number of genes with a PCC of >0.4 across the breast cancer subtypes in the TCGA-breast cohort. **c**, Box plots of the top 1,000 PCCs for the best predicted genes across the breast cancer clinical subtypes in the TCGA-breast cohort.

**Supplementary Fig. 5: Correlation between number of well-predicted genes and chemotherapy treatment response prediction performance.** **a–b**, Scatterplot and linear regression of the AUCs of each cell type-specific treatment response predictor in TransNEO cross-validation cohort (**a**) and IMPRESS external validation cohort (**b**) versus the number of well-predicted genes (PCC > 0.4) for that cell type in the initial TCGA-breast cohort.

**Supplementary Fig. 6: Independence of SLIDE-EX cell type-specific response classifiers from conventional clinicopathologic factors. a,** Forest plots displaying odds ratios (ORs) and 95% confidence intervals from multivariable logistic regression predicting pathological complete response (pCR) in the TransNEO HER2-negative cohort (*n* = 93). Each panel shows results from a separate regression for a given cell type (or bulk expression), with four covariates entered simultaneously: SLIDE-EX classifier prediction (predicted responder vs. predicted non-responder), histologic grade (Grade 3 vs. 2), clinical stage (Stage II vs. III), and nodal status (node-positive vs. node-negative). ORs are displayed on a logarithmic scale; the vertical dashed line indicates OR = 1 (no association). Comparisons are oriented such that OR > 1 indicates higher odds of pCR in the favorable clinical group. P-values from logistic regression are shown to the right of each forest plot. **b**, Bar plots showing AUC values for pCR prediction stratified by histologic grade (top), nodal status (middle), and clinical stage (bottom). Sample sizes for each subgroup are indicated in the legend. The horizontal dashed line indicates AUC = 0.5 (chance-level performance). Stage I (*n* = 2) was excluded due to insufficient sample size for reliable AUC estimation.
